# Independent factors associate with hospital mortality in patients with acute exacerbation of chronic obstructive pulmonary disease requiring intensive care unit admission: Focusing on the eosinophil-to-neutrophil ratio

**DOI:** 10.1371/journal.pone.0218932

**Published:** 2019-07-10

**Authors:** Pei-Ku Chen, Yi-Han Hsiao, Sheng-Wei Pan, Kang-Cheng Su, Diahn-Warng Perng, Hsin-Kuo Ko

**Affiliations:** 1 Department of Chest Medicine, Taipei Veterans General Hospital, Taipei, Taiwan, ROC; 2 School of Medicine, National Yang-Ming University, Taipei, Taiwan, ROC; 3 Institute of Physiology, School of Medicine, National Yang-Ming University, Taipei, Taiwan, ROC; 4 Institute of Public Health, Institute of Physiology, School of Medicine, National Yang-Ming University, Taipei, Taiwan, ROC; National and Kapodistrian University of Athens, GREECE

## Abstract

**Background:**

Factors associated with hospital mortality are unclear in patients with acute exacerbation of COPD (AECOPD) requiring intensive care unit (ICU) admission. We aimed to characterize these patients and identify factors associated with hospital mortality.

**Patients and methods:**

We used a retrospective observational case-control design and recruited patients between January 2015 and March 2017. Of 146 patients enrolled, 24 (16.4%) died during their hospital stay, while 122 survived.

**Results:**

Multivariate logistic regression analyses revealed factors associated with hospital mortality: age (adjusted odds ratio [AOR] 1.12, 95% CI: 1.03–1.23), C-reactive protein (CRP) level >7.5 mg/dL at the emergency room (AOR 4.52, 95% CI: 1.27–16.04), peak eosinophil-to-neutrophil ratio (ENR)×10^2^ on days 8–14 of treatment (AOR 0.22, 95% CI: 0.08–0.63), and in-hospital complications (AOR 4.23, 95% CI: 1.12–15.98) (all *P*<0.05). After receiver operating characteristic curve analyses, cutoff level for peak ENR×10^2^ was 0.224. To examine the synergistic effects of CRP level and peak ENR, we divided patients into four groups: (G0, reference group) Peak ENR×10^2^ >0.224 on days 8–14 and initial CRP <7.5 mg/dL; (G1) Peak ENR×10^2^ >0.224 on days 8–14 and initial CRP >7.5 mg/dL; (G2) Peak ENR×10^2^ <0.224 on days 8–14 and initial CRP <7.5 mg/dL; and (G3) Peak ENR×10^2^ <0.224 on days 8–14 and initial CRP >7.5 mg/dL. For G2 and G3 patients, the AOR of mortality was significantly different from that of the reference group (G2: AOR 10.00, *P* = 0.020; G3: AOR 61.79, *P*<0.001). The relationship between 28-day mortality and the four groups was statistically significant (log-rank test, *P*<0.001).

**Conclusion:**

Older age, initial CRP >7.5 mg/dL, peak ENR on days 8–14, and in-hospital complications were associated with hospital mortality in patients with AECOPD requiring ICU admission. Patients with both biomarkers, initial CRP >7.5 mg/dL, and peak ENR×10^2^ <0.224 on days 8–14 of treatment, had an increased risk of hospital mortality.

## Introduction

Chronic obstructive pulmonary disease (COPD), a common respiratory disease characterized by persistent airflow limitation, is a leading global cause of morbidity and mortality [1]. Acute exacerbation of COPD (AECOPD) is an important event in the management of COPD which negatively impacts health status, rates of hospitalization, and disease progression [2]. Patients with AECOPD who require intensive care unit (ICU) admission for respiratory distress and critical illness [3] face a high mortality rate of 16.9 to 48.8% [4–6]. Clinically, information on patients with AECOPD admitted to the ICU is limited and independent factors to predict their hospital mortality are not routinely available. Therefore, there is an urgent need to identify the factors which predict outcomes in AECOPD patients upon presentation in an emergency room (ER) or upon ICU admission to assist with decisions regarding the early escalation of care, appropriateness of end-of life care, and suitability for early, supported hospital discharge.

The pathophysiology of AECOPD is heterogeneous, and both, exacerbation of the inflammatory cellular endotypes of neutrophils/eosinophils and biological bacterial/viral exacerbation have been emphasized with regard to targeting airway inflammation/infection during treatment of AECOPD patients [7–11]. In a large prospective cohort study of unselected admissions, Steer et al. found that MRC dyspnea scores, eosinopenia, consolidation, acidemia, and atrial fibrillation (DECAF scores) were independent predictors of hospital mortality in patients with AECOPD [12]. Furthermore, in patients hospitalized for AECOPD, Kang et al. reported that patients with neutrophilic exacerbation developed significantly worse hospital outcomes, including ICU admissions and hospital mortality, than those with eosinophilic exacerbation [9]. Based on the current literature, [4, 12–14] both biological characteristics and inflammatory biomarkers may simultaneously impact hospital outcomes and independently predict hospital mortality among AECOPD patients admitted to the ICU.

The aim of the present study was to investigate factors associated with hospital mortality in AECOPD patients requiring ICU admission. We evaluated these patients’ clinical characteristics and inflammatory biomarkers, then determined corresponding risks of hospital mortality.

## Methods

### Study design

The present study utilized a retrospective observational case-control design and was conducted in the 35-bed ICU in the Department of Chest Medicine at Taipei Veteran General Hospital, a 3000-bed tertiary medical center in northern Taiwan. The study was approved by the Taipei Veterans General Hospital’s Institutional Ethical Review Board (VGHTPE-IRB No. 2018-01-013CC). The requirement for informed consent was waived by the IRB based on the institutional guidelines for a retrospective observational study. During the study period, four board certified pulmonologists and critical care physicians provided patient care. Electronic medical records and charts of patients who were admitted to the ICU between January 2015 and March 2017 were reviewed.

### Patients

Patients with a diagnosis of COPD prior to ICU admission and who were admitted to the ICU for AECOPD were enrolled. Exclusion criteria were as follows: 1) admission for non-COPD medical diseases, 2) pulmonary function testing indicating non-obstructive lung disease, 3) no data on pulmonary function test to confirm a diagnosis of COPD, and 4) a stable COPD diagnosis upon ICU admission (n = 23).

Data used in the present study were extracted from medical charts and electronic medical records and included baseline characteristics, data of pulmonary function test prior to ICU admission, medication history, acute Physiology and Chronic Health Evaluation (APACHE) II scores on ICU admission [15], any history of invasive mechanical ventilator (IMV) or non-invasive positive pressure ventilator (NIPPV) use, length of ICU stay, and length of hospitalization. When patients developed new-onset fever or systemic inflammatory response syndrome while in the hospital, surveys were performed to determine the source of infection.

Laboratory data prior to ER visit and during ER and ICU admission were reviewed and recorded. The percentage and counts of eosinophils and neutrophils in the peripheral blood were recorded; further, peak levels of blood eosinophils and neutrophils as well as percentages of each on days 0–2, 3–7, and 8–14 following the patients’ ER visits were recorded. The primary outcome measurement was patient status upon hospital discharge. Enrolled patients were divided into survival and non-survival (hospital mortality) groups.

### Definitions

A COPD diagnosis was made based on spirometry evidence of persistent airflow limitation with a post-bronchodilator FEV_1_/FVC ratio <0.7 [3]. AECOPD was defined as an aggregation of symptoms including increased mucus production and sputum purulence and increased coughing and wheezing that results in a need for additional therapeutics including short acting bronchodilators, antibiotics, and systemic steroids [3]. Eosinophilic exacerbation was defined as blood eosinophil count >2% [8]. Neutrophilic exacerbation was defined as blood leukocyte counts >11000/μL or neutrophils >65%. When a case met both neutrophilia (leukocyte count >11000/μl or neutrophils >65%) and blood eosinophil count >2%, it was classified as eosinophilic exacerbation [9]. In-hospital complications were defined as clinical events including hospital-acquired pneumonia (HAP), urinary tract infections, acute kidney injury (AKI), cardiovascular events (e.g., arrhythmia and myocardial infarction), gastrointestinal events (e.g., gastrointestinal bleeding and ischemic bowel), and stroke. AKI was defined according to the “Kidney Disease Improving Global Outcomes (KDIGO)” guidelines [16].

### Statistical analyses

Kolmogorov-Smirnov testing was used to check the distribution of continuous variables. Continuous variables were described as means (±standard deviation, SD) or medians (interquartile ranges, IQRs), as appropriate. Categorical variables were described as percentages. Chi-square or Fisher’s exact tests were used to compare percentages. Mann-Whitney U tests were used to compare continuous variables with non-normal distributions.

The optimal cut-off value to create dichotomous variables was determined using receiver operating characteristic (ROC) curve analyses. ROC curve analyses were used to check the optimal peak level of eosinophil percentage on days 8–14, peak eosinophil count on days 8–14, peak level of neutrophil percentage on days 8–14, peak neutrophil count on days 8–14, and eosinophil-to-neutrophil ratio (ENR)×10^2^ on days 8–14 (all P-values <0.05). We selected the largest area under the curve (AUC) for univariate analyses. Variables with significant differences (P <0.05) were associated with mortality on univariate analysis and were included in multivariate logistic regressions. A backward elimination procedure was employed to select variables for retention in the final simplified multivariate model. A variable was removed when its removal would cause a change in the exposure odds ratios (ORs) of <10% at each stage of the backward elimination procedure. We considered a two-tailed *P*-value of <0.05 to be statistically significant and ORs with 95% confidence intervals (CIs) were calculated. We analyzed 28-day mortality by Kaplan–Meier analysis and log–rank test. Statistical analyses were performed using SPSS version 20.0 (SPSS, Chicago, IL, USA).

## Results

### Participant characteristics

**Fig 1:**
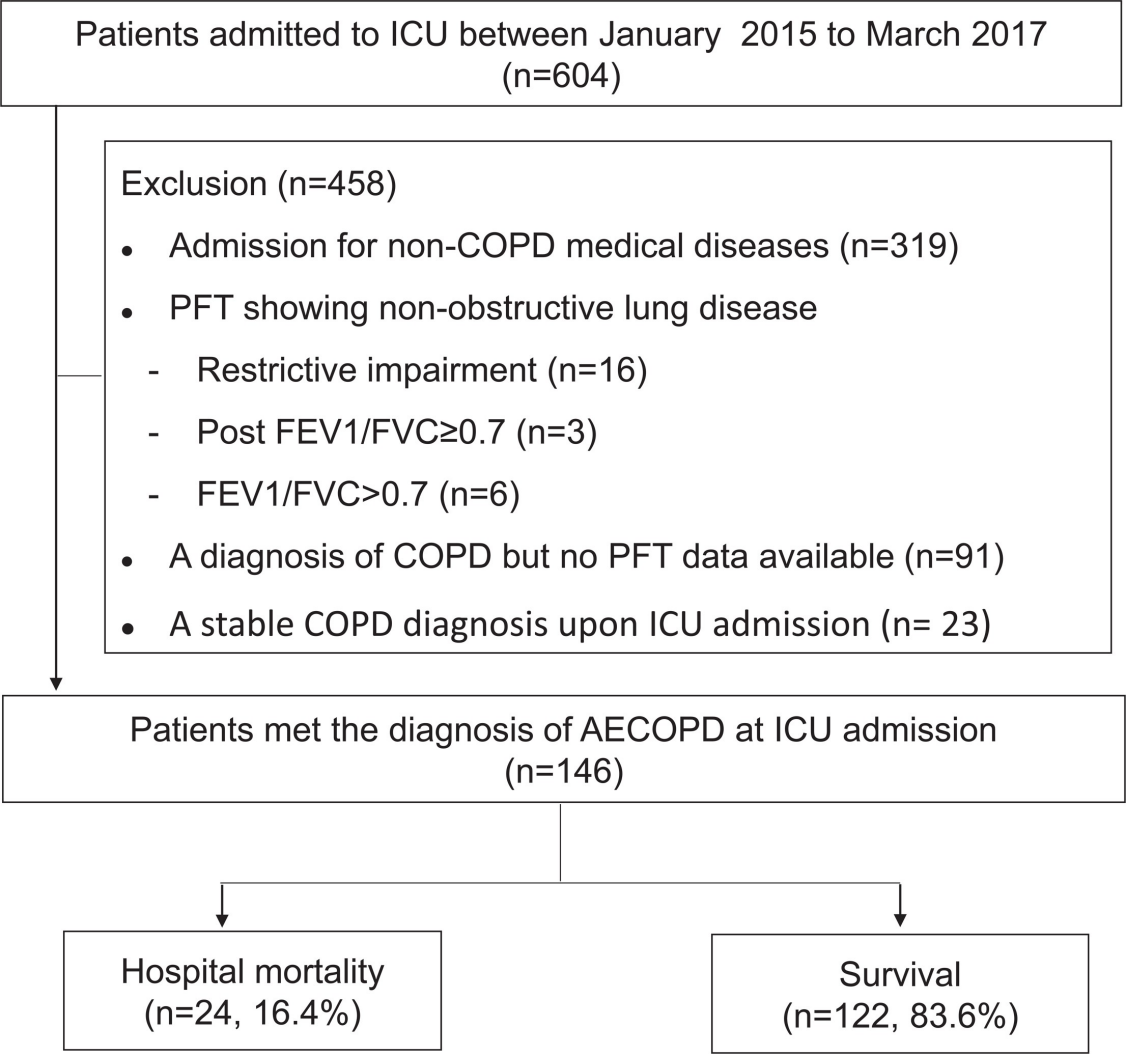
Study flowchart. Abbreviation: FEV1, forced expiratory volume in one second; FVC, forced vital capacity.

Records of a total of 604 consecutive patients admitted to the ICU were reviewed during the study period. A total of 458 patients were excluded due to: 1) admission for non-COPD diseases (n = 319), 2) non-obstructive lung disease according to pulmonary function test (PFT) data (n = 25), 3) a diagnosis of COPD without PFT availability (n = 91) 4) and stable COPD upon ICU admission (n = 23) (Fig 1). Finally, 146 patients (126 men, 20 women) were enrolled in this study and 22 (15.1%) and 118 (84.3%) were classified as having eosinophilic and neutrophilic exacerbations, respectively. The median age and APACHE II scores on ICU admission of study patients were 84 (78–87) years and 15 (11–19), respectively. All patients were >50 years of age; only five patients (3.4%) were <65 years of age. Charlson comorbidity index score was 3 (2–5). The three most common comorbidities were hypertension, gastroesophageal reflux disease, and congestive heart failure. The medium value of post-bronchodilator FEV1/FVC ratio, FEV1 and FEV1%predicted were 47.5% (37.0–59.3), 0.83 L (0.63–1.19) and 42.0%predicted (32.0–60.0), respectively. Twenty-four patients (16.4%, 24/146) used a home ventilator (IMV or NIPPV). At ICU admission, there were 48 patients (32.9%) on IMV and 86 patients (58.9%) on NIPPV. There were 70 (47.9%) patients coexisted of pneumonia on admission. The treatment strategy with the administration of both antibiotic and systemic steroid for AECOPD at ER visit and during ICU admission were 51.4% and 89.7%, respectively. The hospital mortality rate was 16.4% (24/146). None of the patients died within the first week. Four patients died between days 8–14 and another 20 patients died after 2 weeks.

**Fig 2:**
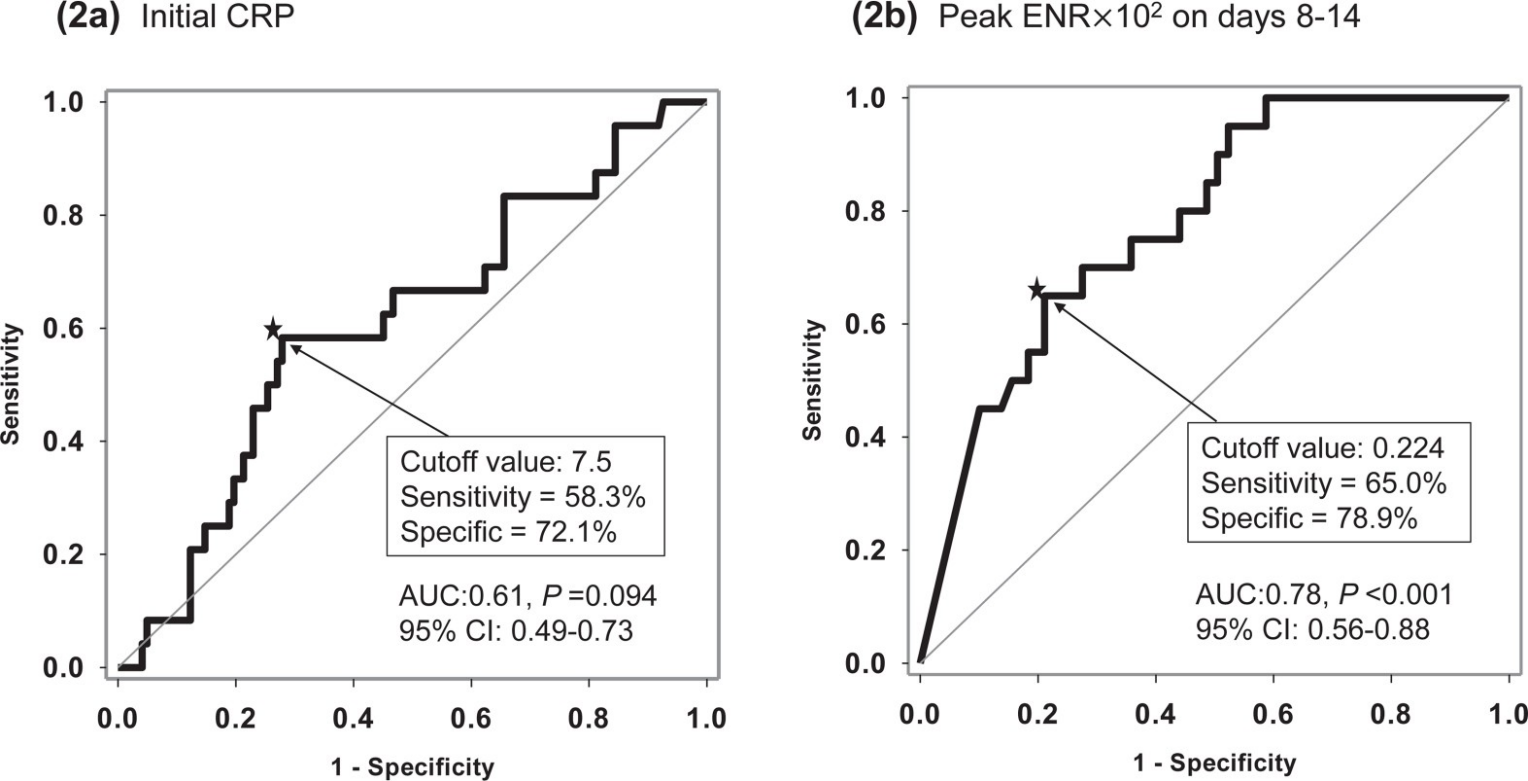
Receiver operating characteristic (ROC) curve for (a) C-reactive protein (CRP) (b) Peak eosinophil-to-neutrophil ratio (ENR)×10^2^ on days 8–14.

Study patients were divided into mortality (n = 24) and survival (n = 122) groups. The baseline characteristics and clinical features at ER admission and during ICU admission in both groups are summarized in Tables 1 and 2. When compared to patients who survived, those who did not were of older age (87 vs. 83 years, *P* = 0.005), had higher FEV1% predicted values (55.5 vs. 41.7%, *P* = 0.049), higher percentages of CRP >7.5 mg/dL, cut-off from ROC curve (Fig 2A) (58.3 vs. 27.9%, *P* = 0.004**)**, higher APACHE II scores (19 vs. 15, *P* <0.001), lower levels of serum albumin (3.1 vs. 3.4 g/dL, *P* = 0.031), and lower pH levels (7.37 vs. 7.41, *P* = 0.045) on ICU admission. Those who died also had higher rates of in-hospital complications (83.3 vs. 42.6%, *P* <0.001), HAP (43.5 vs. 23.0%, *P* = 0.040), AKI (20.8 vs. 2.5%, *P* = 0.003), and stroke (8.3 vs. 0%, *P* = 0.026).

**Table 1:** Baseline characteristics of study patients (n = 146).

	All (n = 146)	Mortality (n = 24)	Survival (n = 122)	*P*-value
Age (years)	84(78–87)	87(83–92)	83(77–87)	0.005
Male, n (%)	126(86.3)	23(95.8)	103(84.4)	0.198
BMI (kg/m^2^)	20.86±4.57	20.18±4.54	21±4.58	0.426
Smoking history, n (%)	118 (81.4)	20(83.3)	98(81)	1
Comorbidities, n (%)				
hypertension	74(50.7)	8(33.3)	66(54.1)	0.063
GERD	50(35)	6(26.1)	44(36.7)	0.330
CHF	44(30.1)	6(25)	38(31.1)	0.549
Diabetes mellitus	41(28.1)	5(20.8)	36(29.5)	0.387
CAD	39(26.7)	7(29.2)	32(26.2)	0.766
Vascular disease	39(26.7)	8(33.3)	31(25.4)	0.423
Arrhythmia	31(21.2)	4(16.7)	27(22.1)	0.550
CKD	23(15.8)	6(25)	17(13.9)	0.218
Charlson comorbidity index	3(2–5)	3(2–4)	3(2–5)	0.380
Charlson comorbidity, n(%)				0.265
0–2	22(15.0)	1(4.2)	21(17.2)	
2–3	68(46.6)	14(58.3)	54(44.3)	
≥ 4	56(38.4)	9(37.5)	47(38.5)	
Bed ridden status (n = 142)	41(28.9)	8(33.3)	33(28.0)	0.597
Eosinophilic exacerbation	22(15.1)	2(8.3)	20(16.4)	0.532
Neutrophilic exacerbation	118(84.3)	20(90.9)	98(83.1)	0.527
PFT (post-bronchodilator)				
FEV1/FVC (%)	47.5(37.0–59.3)	53(41.5–62.3)	46(36.8–59)	0.343
FEV1 (L)	0.83(0.63–1.19)	1.04(0.66–1.36)	0.81(0.62–1.13)	0.132
FEV1 (%predicted)	42.0(32.0–60.0)	55.5(35.0–78.3)	41.7(31.8–59)	0.049
COPD medications, n (%)				
No medication	24(17.4)	4(17.4)	20(17.4)	1.000
Triple	50(36.2)	7(30.4)	43(37.4)	0.526
LABA+LAMA	12(8.7)	1(4.3)	11(9.6)	0.690
LABA+ICS	26(18.8)	5(21.7)	21(18.3)	0.771
LAMA	14(10.1)	5(21.7)	9(7.8)	0.059
Short-acting bronchodilator	12(8.7)	1(4.4)	11(9.6)	0.690
Oral steroid	31(21.5)	6(25.0)	25(20.8)	0.650
Home ventilator use (IMV or NIPPV)	24(16.4)	3(11.5)	21(17.5)	0.570

*Abbreviations*: BMI, body mass index; GERD, gastroesophageal reflux disease; CHF, congestive heart failure; CAD, coronary artery disease, CKD, chronic kidney disease; PFT, pulmonary function test; FEV1, forced expiratory volume in one second; FVC, forced vital capacity; LABA, long acting beta agonist; LAMA, long acting muscarinic antagonist; ICS, inhaled corticosteroid; IMV, invasive mechanical ventilation; NIPPV, noninvasive positive pressure ventilation

**Table 2:** Clinical features at ER and during ICU admission in study patients.

	All (n = 146)	Mortality (n = 24)	Survival (n = 122)	*P*-value
**ER visit**				
PH	7.35(7.28–7.42)	7.35(7.28–7.39)	7.35(7.28–7.43)	0.643
PaCO2 (mmHg)	53(41–67)	50(38–75)	54.0(42–65)	0.843
PaO2/FiO2 (mmHg)	206(136–326)	189(119–358)	207(139–309)	0.727
CRP (mg/dL)	4.0(0.9–9.4)	8.1(1.2–14.1)	3.4(0.8–8.5)	0.094
CRP>7.5 mg/dL, n(%)	48(32.0)	14(58.3)	34(27.9)	0.004
NT-proBNP (n = 110)	1218(369–4783)	2295(471–6159)	1012(331–4706)	0.136
Inotrope use, n(%)	15(10.3)	5(20.8)	10(8.2)	0.074
Steroid only	19(13.0)	2(8.3)	17(13.9)	0.740
Antibiotic only	38(26.0)	7(29.2)	31(25.4)	0.701
Steroid+ antibiotics	75(51.4)	11(45.8)	64(52.5)	0.533
**ICU admission**				
Pneumonia, n(%)	70(47.9)	14(58.3)	56(45.9)	0.256
APACHE II	15(11–19)	19(14–22)	15(11–18)	<0.001
Albumin (g/dL)	3.3(2.9–3.7)	3.1(2.8–3.3)	3.4(3.0–3.7)	0.031
Creatinine (mg/dL)	1.16(0.9–1.40)	1.33(0.86–2.33)	1.09(0.9–1.34)	0.113
PH	7.40(7.33–7.47)	7.37(7.28–7.41)	7.41(7.34–7.48)	0.045
PaCO2 (mmHg)	48(37–62)	48(35–63)	48(37–61)	0.939
PaO2/FiO2 (mmHg)	254(178–323)	195(124–311)	258(185–325)	0.139
IMV use, n(%)	48(32.9)	8(33.3)	40(33.1)	0.979
NIPPV use, n(%)	86(58.9)	14(58.3)	72(59)	0.950
Steroid only	2(1.4)	1(4.2)	1(0.8)	0.303
Antibiotic only	13(8.9)	1(4.2)	12(9.8)	0.695
Steroid+ antibiotics	131 (89.7)	22(91.7)	109 (89.3)	1.000
Steroid use via intravenous route for 1wk	49 (34.5)	7 (30.4)	42 (35.3)	0.654
**Hospital Complications, n(%)**	72(49.3)	20(83.3)	52(42.6)	<0.001
HAP	38(26.2)	10(43.5)	28(23.0)	0.040
UTI	16(11)	4(16.7)	12(9.8)	0.302
AKI	8(5.5)	5(20.8)	3(2.5)	0.003
CV event	8(5.5)	2(8.3)	6(4.9)	0.618
GI event	14(9.6)	3(12.5)	11(9)	0.703
Stroke	2(1.4)	2(8.3)	0(0)	0.026
Infections newly diagnosed on days 8–14	29(21.0)	7(33.3)	22(18.8)	0.150
ICU stay (days)	13(10–19)	13(8–24)	13(10–19)	0.785
Hospital (days)	21(15–35)	20(10–32)	21(15–36)	0.311

*Abbreviation*: CRP, C-reactive protein; NT-proBNP, N-terminal pro-brain natriuretic peptide; IMV, invasive mechanical ventilation; NIPPV, noninvasive positive pressure ventilation; APACH II, acute physiology and chronic health evaluation; HAP, hospital acquired pneumonia; UTI, urine tract infection; AKI, acute kidney injury; CV, cardiovascular; GI, gastrointestinal; ICU, intensive care unit.

### Blood eosinophil and neutrophil counts among surviving and non-surviving patients

The percentage and levels of blood eosinophils and neutrophils prior to patients’ ER visits and their peak levels during days 0–2, days 3–7, and days 8–14 of treatment are shown in Table 3. On days 3–7, the peak percent of blood neutrophils was higher in those patients who passed away than those did not (89.5 vs. 86.6%, *P* = 0.049). On days 8–14, patients who passed away also presented with lower peak percentages and counts of blood eosinophils than those who survived (0.1 vs. 0.8%, *P* <0.001; 12 vs. 95 u/l, *P* <0.001, respectively), as well as higher peak percentages and counts of blood neutrophils (91.1 vs. 80.1%, *P* <0.001; 12.75 vs. 8.66×10^9^/L, *P* <0.008, respectively). The ENR×10^2^ on days 8–14 was lower in patients who passed away than those who did not (0.114 vs. 0.872, *P* <0.001).

**Table 3:** The percentage and count of blood eosinophil and neutrophil prior to ER visit and during admission in the study patients.

	All	Mortality	Survival	*P*-value
**Prior to ER visit**	**(n = 146)**	**(n = 24)**	**(n = 122)**	
Eosinophil (%)	2.3(1.1–3.8)	1.8(0.9–3.6)	2.6(1.2–3.9)	0.279
Eosinophil (×10^9^/L)	0.19(0.08–0.30)	0.13(0.06–0.25)	0.20(0.09–0.32)	0.147
Neutrophil (%)	68.1(61.4–76.7)	68.1(57.5–79.8)	68.1(63.6–76.3)	0.662
Neutrophil (×10^9^/L)	5.24(4.02–6.90)	5.21(3.70–5.93)	5.26(4.13–6.94)	0.504
ENR×10^2^	3.53(1.67–6.40)	2.61(1.27–6.32)	3.66(1.82–6.42)	0.374
**Days 0–2**	**(n = 146)**	**(n = 24)**	**(n = 122)**	
Eosinophil peak %	0.4(0–1.4)	0.3(0–1.1)	0.4(0.1–1.5)	0.214
Eosinophil peak (×10^9^/L)	0.04(0–0.14)	0.02(0–0.13)	0.05(0.01–0.15)	0.158
Neutrophil peak %	83.3(75.9–87.4)	83.7(78.6–88.6)	82.6(75.2–87.3)	0.570
Neutrophil peak (×10^9^/L)	8.56(6.19–12.87)	8.19(5.69–12.91)	8.64(6.41–12.87)	0.460
ENR×10^2^	0.458(0–2.159)	0.356(0–1.328)	0.458(0.104–2.252)	0.235
**Days 3–7**	**(n = 144)**	**(n = 24)**	**(n = 120)**	
Eosinophil peak %	0.1(0–0.6)	0(0–0.5)	0.1(0–0.7)	0.158
Eosinophil peak (×10^9^/L)	0.01(0–0.07)	0(0–0.04)	0.01(0–0.08)	0.164
Neutrophil peak %	86.8(79.7–91.3)	89.5(81.4–94.3)	86.6(77.6–91.0)	0.049
Neutrophil peak (×10^9^/L)	8.59(6.46–12.46)	10.53(6.57–12.93)	8.43(6.46–12.40)	0.218
ENR×10^2^	0.113(0–0.736)	0(0–0.590)	0.115(0–0.878)	0.151
**Days 8–14**	**(n = 129)**	**(n = 20)**	**(n = 109)**	
Eosinophil peak %	0.6(0.2–1.8)	0.1(0–0.6)	0.8(0.2–2.0)	<0.001
Eosinophil peak (×10^9^/L)	0.07(0.02–0.18)	0.01(0–0.06)	0.10(0.03–0.22)	<0.001
Neutrophil peak %	83.1(72.1–89.0)	91.1(85.0–94.0)	80.1(71.4–87.4)	<0.001
Neutrophil peak (×10^9^/L)	9.09(6.72–13.47)	12.75(8.53–17.03)	8.66(6.13–12.59)	0.008
ENR×10^2^	0.761(0.167–2.411)	0.114(0–0.686)	0.872(0.244–2.575)	<0.001

*Abbreviation*: ENR, eosinophil-to-neutrophil ratio

### Independent factors associated with mortality

**Fig 3:**
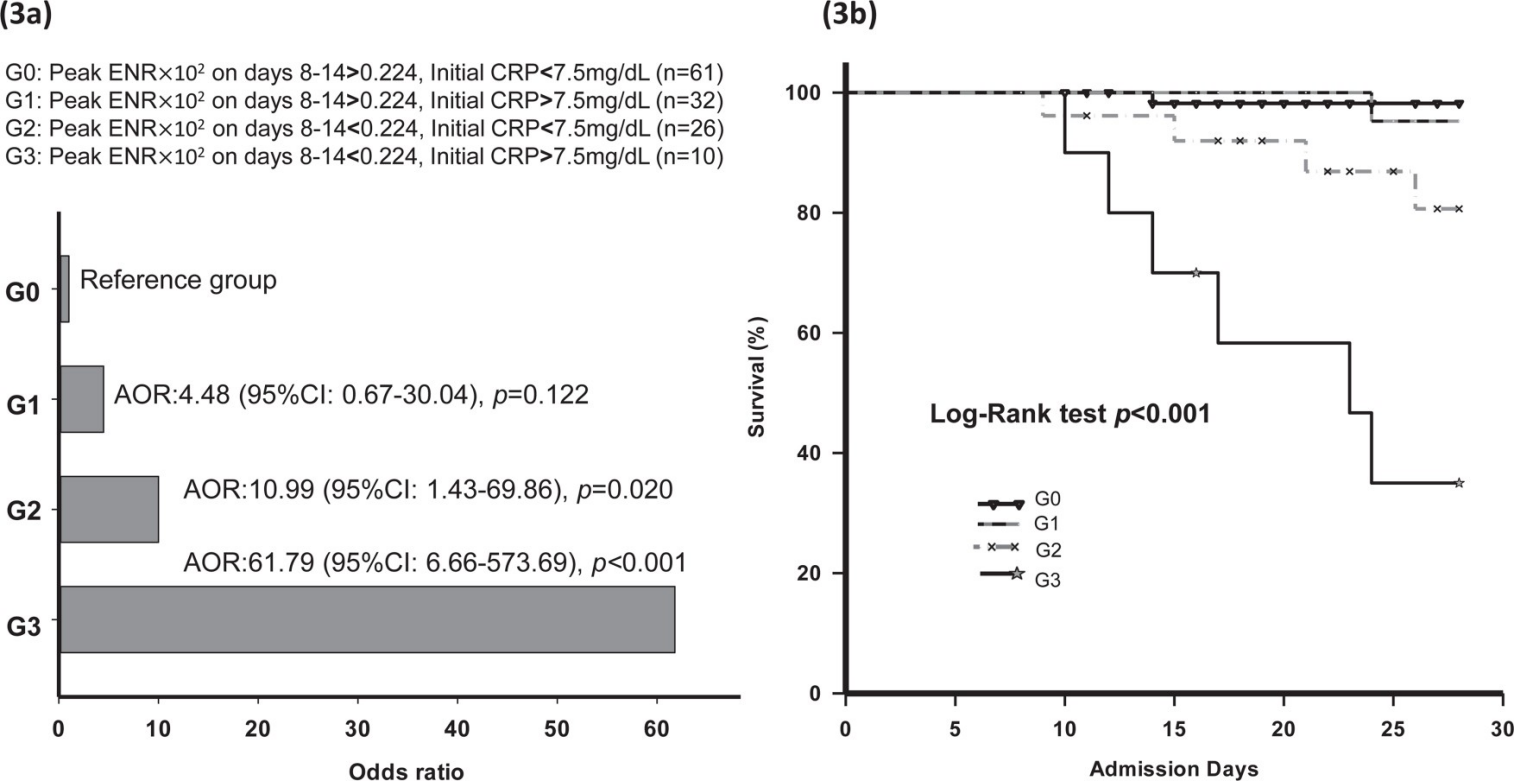
**(a)** The synergistic impact of initial CRP and peak ENR on days 8–14 of treatment on the risk of hospital mortality. The adjusted odds ratios (AOR) with 95% confidence intervals (95% CIs) for mortality are shown. **(b)** Kaplan–Meier curves for 28-day mortality in each group.

Multivariate logistic regression analyses revealed independent risk factors associated with hospital mortality including age (adjusted odds ratio [AOR] 1.12, 95% CI: 1.03–1.23, *P =* 0.011), initial CRP >7.5 mg/dL at ER (AOR 4.52, 95% CI: 1.27–16.04, *P =* 0.020), peak ENR×10^2^ levels on days 8–14 (AOR 0.22, 95% CI: 0.08–0.63, *P =* 0.005), and in-hospital complications (AOR 4.23, 95% CI: 1.12–15.98) (Table 4). Age, CRP >7.5 mg/dL and peak ENR×10^2^ level on days 8–14 of treatment were still statistically significant, after adjust sex, BMI and intravenous steroid use on days 7. The cutoff level for ENR×10^2^ on days 8–14 was analyzed by using a ROC curve in differentiating hospital mortality versus survival patients and the values was 0.224 (AUC = 0.78, sensitivity = 65.0%, specificity = 78.9%, *P* <0.001, 95% CI: 0.68–0.88) (Fig 2B). Cox regression was performed for examining the predictive effect for mortality via admission CRP level and peak level of ENR×10^2^ on days 8–14 of treatment. Patients with peak ENR×10^2^ >0.224 on days 8–14 and initial CRP<7.5mg/dL comprised the reference group (G0). Patients with peak ENR×10^2^ >0.224 on days 8–14 and initial CRP >7.5mg/dL were in group 1 (G1). Patients with peak ENR×10^2^ <0.224 on days 8–14 and initial CRP <7.5mg/dL were in group 2 (G2). Patients with peak ENR×10^2^ <0.224 on days 8–14 and initial CRP >7.5mg/dL were in group 3 (G3). For G2 and G3 patients, the AOR of mortality was significantly different from that of the reference group (G2: AOR 10.00, 95% CI: 1.43–69.86, *P* = 0.020; G3: AOR 61.79, 95% CI: 6.66–573.69, *P*<0.001) (Fig 3A). The relationship for 28-day mortality and the four groups was statistically significant. (long-rank test *P*<0.001) (Fig 3B).

**Table 4:** Univariate and multivariate logistic regression analysis of risk factors for hospital mortality in the study patients (n = 146).

Variable	Univariate		Simplified model using backward LR method ^a^	
	OR(95%CI)	*p*	AOR(95% CI)	*p*
Age	1.09(1.02–1.17)	0.009	1.12(1.03–1.23)	0.011
Sex	4.24(0.54–33.32)	0.169	_^a^	
BMI	0.96(0.87–1.06)	0.424	_^a^	
APACHE II	1.16(1.07–1.26)	<0.001	_^a^	
Albumin	0.42(0.18–0.99)	0.048	_^a^	
In-hospital complications	6.73(2.17–20.88)	0.001	4.23(1.12–15.98)	0.033
Initial CRP>7.5 mg/dL	3.62(1.47–8.94)	0.005	4.52(1.27–16.04)	0.020
PH at ICU admission	0.01(0–1.34)	0.065		
Peak ENR×10^2^ on days 8–14	0.28(0.11–0.72)	0.008	0.22(0.08–0.63)	0.005
Peak Neutrophil % on days 8–14	1.14(1.06–1.23)	0.001		

^a^ Variables entered into multivariate logistic regression analysis with backward elimination method did not retain in the final model.

*Abbreviations*: BMI, body mass index; APACH II, acute physiology and chronic health evaluation; CRP, C-reactive protein; ICU, intensive care unit; ENR, eosinophil-to-neutrophil ratio.

## Discussion

AECOPD is critical to the management of COPD because it negatively impacts health status, rates of hospitalization, and disease progression [2]. A variety of the inflammatory cells and molecular mediators are involved in the pathophysiology of AECOPD [17, 18]. In the current study, we evaluated biological characteristics and inflammatory endotypes associated with mortality in patients with AECOPD. We then identified age, CRP, and peak levels of ENR×10^2^ on days 8–14 of treatment, in-hospital complications as independent factors associated with hospital mortality. Additionally, patients with both biomarkers, initial CRP>7.5 mg/dL and peak ENR×10^2^ on days 8–14 <0.224 of treatment, had an increased risk for hospital mortality. Our results suggest that factors in differential timing (age and CRP at admission, ENR during the first 2 weeks, and in-hospital complications) could be associated with hospital mortality in patients with AECOPD requiring ICU admission.

Some clinical characteristics have been demonstrated to predict short- and long-term outcomes among COPD patients hospitalized for disease exacerbation. Variables associated with mortality after AECOPD reflect underlying severity of acute illness [19]. Steer et al. reported that dyspnea, acidemia, and atrial fibrillation are independent predictors of hospital mortality in patients hospitalized for AECOPD [12]. In patients with AECOPD and who were admitted to the ICU, Ongel et al. reported comorbidities (coronary artery disease, arrhythmia, and hypertension) and clinical variables (IMV support, BMI <20 kg/m^2^, pneumonia, chronic hypoxia, and high APACHE II scores) upon ICU admission that were predictive of ICU mortality [5]. Spannella et al. demonstrated that preadmission functional dependence, cognitive impairment, corticosteroid use and elevated NT-proBNP at admission were risk factors for mortality in the oldest patients [20]. In the current study, we similarly evaluated the clinical characteristics/variables and inflammatory endotypes present in AECOPD patients admitted to the ICU to reveal that age, initial CRP, and peak levels of ENR on days 8–14 of treatment were independent factors predictive of mortality. To our knowledge, our study is the first to demonstrate that specific clinical characteristics and inflammatory endotypes associated with mortality in patients with AECOPD requiring ICU admission. Compared with previous studies in which ICU mortality ranged from 16.9 to 48.8% [4–6] and hospital mortality ranged from 11% to 50% [6, 19, 21, 22], the mortality rates in our study (16.4%) are relatively low. This is likely to be related to lower APACH II score (median, 15) at ICU admission in our study patients. In addition, improvement of acute care and management of chronic illness may have contributed to the relatively low mortality rate.

AECOPD is commonly classified based on eosinophilic and non-eosinophilic exacerbation and the impact of eosinophils and neutrophils on patient outcomes has been clarified in several studies [9, 23, 24]. High blood eosinophil is related to higher sputum eosinophil, BAL (bronchoalveolar lavage fluid) eosinophil and submucosal eosinophil [25]. In the SPIROMICS cohort, blood eosinophil counts association with sputum eosinophil counts were also noted [25, 26] Therefore, blood eosinophil counts may serve as a surrogate for sputum eosinophilic airway inflammation in AECOPD [7] and their levels on admission in severe COPD exacerbation cases predict readmission rates [19]. For instance, Salturk et al. reported better outcomes with eosinophil levels >2% in AECOPD with acute respiratory failure requiring ICU admission [24]. Additionally, eosinophils might have anti-bacterial properties found via mouse study [27]. Eosinophil activation also be found at the primary infection with viruses in the lungs in response to human rhinovirus and respiratory syncytial virus [28, 29]. Therefore, eosinopenia is reported to be associated with higher rate of hospital mortality in AECOPD cases [12]. Eosinopenia may thus be a helpful clinical indicator of non-infection or infection and SIRS/infection in ICU patients [30].

In addition to eosinopenia, patients with neutrophilic exacerbations may experience significantly worse clinical outcomes and increased ICU admission [9]. In the present study, we found that the endotypes of eosinophilic and neutrophilic exacerbation on ICU admission were not predictive of hospital mortality. We analyzed percentages and counts of eosinophils and neutrophils in the first 2 weeks during hospitalization, then combined these two critical biomarkers to determine ENR, which comprised an independent factor associated with hospital mortality in patients with AECOPD requiring ICU admission. No existing studies have shown that ENR during hospitalization, especially on days 8–14 of treatment, constitute a factor associated with hospital mortality in patients with AECOPD. The present study may remind intensivists to remain aware of the potential influence of inflammatory endotypes on patient outcomes in this patient population. A higher proportion of newly diagnosed infections on days 8–14 was noted in the mortality group, compared to that in the survival group (33.3% vs. 18.8%); however, that difference was not statistically significant in our study patients. A low level of eosinophils with a high level of neutrophils in patients who have died may be the result of bacterial infection. A variety of treatments, such as steroid administration and antibiotic use might also affect ENR. Patients with persistent low ENR after treatment may experience a relatively poor prognosis.

Additionally, our study demonstrated that an initial CRP >7.5 mg/dL (evaluated at ER admission) was an independent factor associated with hospital mortality after ICU admission in AECOPD patients. CRP is a useful biomarker for the confirmation of AECOPD in patients with dyspnea, increasing sputum volume, and purulence [31]. High CRP levels were also noted in patients with non-eosinophilic AECOPD [13] and may be associated with treatment failure [32]. This may result from the baseline severity of COPD with superimposed bacteria-associated exacerbation [7, 33] and be further associated with poor prognoses in these patients [33]. Another factor associated with hospital mortality was in-hospital complications, which also have affected the duration of ICU stay and shortened the duration of hospital stay.

Corticosteroids induce eosinopenia and neutrophilia in clinical practice [34, 35]. In the current study, nearly 90% of enrolled patients were treated with steroids upon ICU admission and 34% received steroids intravenously for one week. This administration of corticosteroids prior to ER admission and during hospitalization may serve a role in changing eosinophil and neutrophil levels upon ER admission and during ICU admission. Therefore, we adjusted the influence of systemic steroids per a multivariate logistic regression analysis and reported the use of ENR on days 8–14 as an independent factor associated with hospital mortality in patients with AECOPD requiring ICU admission.

While it provides some critical conclusions, the present study has several limitations that warrant discussion. First, its retrospective design at a single center, as well as non-standardized protocol, introduced some bias. Additionally, some data might be missing due to recording errors or lapses. Second, our hospital is a tertiary referral center primarily used for the care of military veterans. Therefore, the average age of the study population was relatively high. We did not consider several other confounding factors, such as functional evaluation and activities of daily living assessment, which could affect hospital mortality in geriatric patients [20]. Third, we focused patient recruitment on those with AECOPD who were admitted from the ER to the ICU. We did not examine patients who were admitted to the general ward or discharged from the ED and the factors described here may not be suitable for all patient populations. Furthermore, one of the items (ENR) can be only be measured between days 8–14. But, our result reminded physicians to observe the ENR. The peak ENR×10^2^ on days 8–14 of treatment < 0.224 might indicate patients at high risk of hospital mortality. Fourth, the study was done with few patients. The enormous confidence interval of AOR for the synergistic effect of initial CRP and peak ENR×10^2^ on days 8–14 probably due to the small sample size. Further validation needs to be performed. However, the estimated power was 0.96 for our sample size. Finally, treatments, including initial steroid dose, bronchodilator administration, and the timing of blood tests, differed between patients and clinicians. Future prospective methods for standardization of clinical protocols are required. Future studies which more carefully address specific endotype markers may allow for better detection and classification of patients with AECOPD and understanding of their risk for mortality.

## Conclusion

Our findings revealed that old age, high initial CRP, low peak ENR on days 8–14 of treatment and in-hospital complications were factors associated with hospital mortality among AECOPD patients requiring ICU admission. Particularly, patients with both biomarkers, initial CRP>7.5 mg/dL and peak ENR×10^2^ on days 8–14 <0.224 of treatment, had an increased risk for hospital mortality.

## Supporting information

S1 Dataset Participant raw data. (XLSX)
